# INJURY EPIDEMIOLOGY IN BEACH TENNIS: INCIDENCE AND RISK FACTORS

**DOI:** 10.1590/1413-785220243201e268301

**Published:** 2024-03-22

**Authors:** Fabio Lucas Rodrigues, Paulo Sergio Barone, Ramylla Saldanha Penha, Isabela Pagliaro Franco

**Affiliations:** 1 Centro Universitário Saúde ABC, ABC Medical School Department of Orthopaedics and Traumatology, Santo André, SP, Brazil.

**Keywords:** Wounds and Injuries, Epidemiology, Athletes, Musculoskeletal System, Ferimentos e Lesões, Epidemiologia, Atletas, Sistema Musculoesquelético

## Abstract

**Introduction:**

Due to the growing increase in beach tennis practice in Brazil and the lack of studies on the injuries that occur in this sport, it has become necessary to develop more research on the subject.

**Objective:**

to identify risk and protection factors for injuries in beach tennis, in order to generate prevention strategies for musculoskeletal injuries.

**Method:**

A cross-sectional epidemiological study, level 3 of evidence, was carried out through an electronic form with 698 Beach Tennis players, who answered questions about their relationship with the practice of the sport and occurrences of injuries. We researched the prevalence of injuries, their types, and their relation with personal physical characteristics and the practice of other sports.

**Results:**

We found a positive relationship between injuries when associated with longer exposure time and the presence of a previous injury. We did not find differences regarding BMI, gender, and stretching and muscle strengthening performance.

**Conclusion:**

the most frequent non-traumatic injuries were to the elbow and shoulder (tendonitis) and traumatic (sprain) injuries to the knee and ankle. ***Level of Evidence II; Cohort Study.***

## RESUMO

**Introdução:**

Devido ao crescente aumento da prática de Beach Tennis no Brasil e a carência de estudos sobre as lesões que ocorrem nesse esporte se faz necessário o desenvolvimento de mais pesquisas sobre o tema.

**Objetivo:**

identificar fatores de risco e de proteção para lesões no Beach Tennis, afim de gerar estratégias de prevenção às injurias musculoesqueléticas.

**Método:**

realizado estudo epidemiológico transversal nível 3 de evidência através de um formulário eletrônico com 698 praticantes de Beach Tennis, que responderam questionamentos sobre sua relação com a prática do esporte e ocorrências de lesões. Pesquisamos a prevalência das lesões e seus tipos, assim como sua relação com as características físicas pessoais e prática de outros esportes.

**Resultados:**

encontramos relação de positividade para lesões quando associadas a maior tempo de exposição e presença de lesão prévia. Não encontramos diferença quanto ao IMC, sexo e a realização de alongamento e fortalecimento muscular.

**Conclusão:**

as lesões mais frequentes não traumáticas foram no cotovelo e ombro (tendinite) e traumáticas (entorse) de joelho e tornozelo. ***Nível de Evidência II; Estudo de Coorte.***

## INTRODUCTION

Beach Tennis (BT) was created in 1987 in the province of Ravennana, Italy. In 1996 the sport began to become professionalized. The modality arrived in Brazil in 2008 in the state of Rio de Janeiro and since then the sport has been growing rapidly to other Brazilian cities, including non-coastal cities.^1^

Despite the numerous studies involving injuries that occur in tennis players around the world,^1^ we found available in the literature only one epidemiological study addressing BT players.^2^ This is a cross-sectional epidemiological study that analyzed practitioners of different levels of BT in the French island of Réunion, where the sport was raised to professionalism in the 1990s.^4^

With the recent increase in the practice of BT, especially in Brazil,^1^ the possible injuries associated with its practice are not known. Research to better understand them is important to develop preventive and therapeutic programs, and to minimize their impact, such as discouragement.

The objective of this study is to characterize the BT players, the prevalence of injuries, identify risk and protection factors for sports-related musculoskeletal disorders, and their impact on the player performance.

## METHODS

Cross-sectional epidemiological study in BT players, invited to complete a questionnaire with the use of a virtual application. The participation in this study was voluntary and all individuals who agreed to participate signed an informed consent form to follow up on the application of the questionnaire.

This study was approved by the Research Ethics Committee, CAAE 60292822.5.0000.0082 on August 15, 2022, and does not require CONEP review.

We asked the following information in the questionnaire: identification and anthropometric data (age, height, weight, gender, dominant limb), time of BT practice (over the years, weekly frequency, how many sets), if they do any concomitant physical preparation (Beach Tennis classes, practice of other sports), players’ level (participation in competitions, category); physical injuries: previous or current with the time away, type of injury, affected limb, professional who made the diagnosis, pain during and after the match, and whether stretching or physical strengthening is done before or after training and matches.

With the data obtained we will evaluate the prevalence and relationship of injuries with gender, BMI, frequency of games, practice of other sports, physical conditioning (weight training, stretching, and strengthening), and previous injuries. Also the correlation between the affected area and type of injury.

For these data descriptive and inferential statistical methods will be applied. Qualitative variables will be presented by distribution of absolute and relative frequencies. In the inferential part will be applied: (a) To compare the proportional distribution of nominal variables will be applied the G Test of independence; (b) To evaluate the distribution of the location of the lesion will be applied the Chi-square test of adherence. The alpha error will be previously set at 5% for rejection of the null hypothesis and the statistical processing will be performed in the BioEstat version 5.3 and STATA release 17 programs.

## RESULTS

The data collection time was 30 days, with a total number of 698 participants.

Participants of different ages filled out the survey and we identified that the older the age group, the higher the risk of the patient to suffer an injury, being that among the players under 30 years old 26.10% had injuries, between 30 and 50 years old 51.2%, and over 50 years old 61.8%.

The questionnaire was filled out by a majority of females (67.3%), but this variable does not represent a significant difference for the occurrence of injuries (p-value=0.5871) when compared to males.

In the anthropometric data evaluation, we verified that most of the practitioners (66.8%) present BMI less than 25 kg/m² (normal value). The correlation of the presence of injuries among individuals with BMI below or above 25 also showed no significant difference (p-value=0.1865).

Regarding the time of practice, it was shown that the most common is from 1 to 2 years (28.5%) and that this has a significant difference with the appearance of injuries (p-value<0.0001*), showing also that for players who play from 3 to 5 years the occurrence of injuries is 75%.

When we evaluated the weekly frequency of games, most of the players play from 2 to 3 times a week (40.4%), and this data also presents a significant difference for the occurrence of injuries (p-value=0.0004*), and for players who play from 5 to 7 times a week the occurrence of injuries reaches 66.2%.

Another variable that presented significant difference (p-value<0.0001*) was the time practiced per game with the presence of injury, being that the most common time of game (66.8%) was 2 hours (3 to 5 sets), and a time of game longer than 3 hours is related to 67.1% of injuries. (Table 1)

**Table 1:** Site affected by the injury in n=698 beach tennis practitioners. Year 2022.

Affected location	n	%
Elbow	163	23.4
Shoulder	98	14.0
Knee	82	11.7
Feet	62	8.9
Ankle	62	8.9
Calf	52	7.4
Cervical spine	48	6.9
Lumbar spine	48	6.9
Hand	44	6.3
Fist	44	6.3
Hips	26	3.7

* p-value <0.0001*, Chi-square grip.

We evaluated the relationship between the practice of other sports concomitant to the BT (74.5%) and the occurrence of injuries, obtaining no significant difference (p-value=0.1452) between those who practice or not.

A stretching routine before and after games is not performed in 53.7%, and these had no significant difference with the number of injuries when compared to those who perform (p-value=0.0926).

The practice of physical conditioning and weight training is accomplished by most of the practitioners (73.6%), but it was not shown as a protective factor for injuries, also not presenting significant difference (p-value=0.5926).

There is a positive correlation between the existence of a previous injury and the appearance of a new one (p-value<0.0001*), for of those who had already suffered an injury, 74.9% had a new one in the BT. (Table 2)

**Table 2:** Sex, BMI, Practice time, How many times per week and Playing time according to the presence of injury in n=698 beach tennis practitioners. Year 2022.

	Injury						
	Present		Absent		General		
	n	%	N	%	n	%	p-value
**Sex**							**0.5871**
Female	250	53.2	220	46.8	470	67.3	
Male	127	55.7	101	44.3	228	32.7	
IMC							**0.1865**
Greater than 25	134	57.8	98	42.2	232	33.2	
Less than 25	243	52.1	223	47.9	466	66.8	
**Practice time**							<0.0001*
Less than 1 year	44	34.1	85	65.9	129	18.5	
1 to 2 years	86	43.4	113	57.1	199	28.5	
2 to 3 years	70	55.1	57	44.9	127	18.2	
3 to 5 years	87	75.0	29	25.0	116	16.6	
More than 5 years	90	70.9	37	29.1	127	18.2	
**How many times a week**							0.0004*
1 time	48	38.4	77	61.6	125	17.9	
2 to 3 times	151	53.5	131	46.5	282	40.4	
3 to 5 times	130	59.9	87	40.1	217	31.1	
5 to 7 times	47	66.2	24	33.8	71	10.2	
Not once	1	33.3	2	66.7	3	0.4	
**Playing time**							<0.0001*
Up to 1 hour (Approx. 3 sets)	55	38.5	88	61.5	143	20.5	
Up to 2 hours (Approx. 3 to 5 sets)	263	56.4	203	43.6	466	66.8	
More than 3 hours (More than 6 sets)	57	67.1	28	32.9	85	12.2	
Not informed	2	50.0	2	50.0	4	0.5	

* G test of independence, comparing Presence x Absence of Injury.

When we researched which limbs were most frequently injured we found that with a significant difference (p-value <0.0001*) the elbow was the most affected (23.4%), followed by the shoulder (14.0%).

Among the types of injuries reported we have elbow tendinitis (80.26%) as the most prevalent, followed by knee sprain and contusion (61.90%) and shoulder tendinopathy (60.52%). Excluded from these results the patients who had multiple injuries (20.63%). (Table 3)

**Table 3:** Do you practice another sport, Do you have any stretching/strengthening routine before and after training/games?, Do you do any physical conditioning/bodybuilding work? and Previous injury according to the presence of injury in n=698 beach tennis practitioners. Year 2022.

	Present		Absent		General		p-value
	n	%	n	%	n	%	
**Practice another sport**							**0.1452**
Yes	272	52.3	248	47.7	520	74.5	
No	105	59.0	73	41.0	178	25.5	
**Do you have any stretching/strengthening routines before and after workouts/games?**							**0.0926**
Yes	186	57.6	137	42.4	323	46.3	
No	191	50.9	184	49.1	375	53.7	
**Do you do any fitness/bodybuilding work?**							**0.5908**
Yes	274	53.3	240	46.7	514	73.6	
No	103	56.0	81	44.0	184	26.4	
**Previous injury**							**<0.0001** *
Yes	274	74.9	240	25.1	514	47.9	
No	127	34.9	237	65.1	364	52.1	

* G test of independence, comparing Presence x Absence of Injury.

## DISCUSSION

According to data from the Brazilian Tennis Confederation,^1^ currently the BT is practiced by more than 500 thousand people spread all over the continents, regardless of sex and age. Data from the International Tennis Federation also point out that Brazil is the second biggest force in the world in this sport, second only to Italy, the country that created the modality.^1^

All the questions about the longest time of exposure to the sport presented statistical relevance as risk factors for injury. The longer the time of practice, the higher the risk of injury. Up to 75% of players with 3 to 5 years of practice, 66.2% of those who play 5 to 7 times a week, and 67.1% of those who play for more than 3 hours per game can present injuries. These data match those collected when studying injuries in tennis players,^3^ where training overload generates a cumulative effect, and repetitive micro traumas were one of the main triggering factors for musculoskeletal injuries.

When asked about the places already injured the participants mentioned: cervical spine, lumbar spine, elbow, knee, hand, shoulder, calf, foot, wrist, hip, and ankle, being the most affected limbs the elbow (23.4%), followed by the shoulder (14%). Results were also found in Tennis and Badminton players, because they are racquet sports and have been demonstrated in previous studies with competitive tennis players,^4^ which indicated elbow and shoulder as the most affected joints, followed by the knee.

And as for the most common type of injury, we found data consistent with those shown in the work base of this study,^4^ where tendinopathies are more common in the upper limbs (elbow 80.26%, shoulder 60.52%, and wrist 45.45%), while traumatic injuries such as sprains are more common in the lower limbs (ankle and foot 37.93%), followed by muscle injuries (calf 93.75%).

When we analyzed the relationship between the physical conditioning of the practitioners and the incidence of injuries, we believed that those who did the preparation would have this habit as a factor for preventing injuries, but we did not find the expected results, because there is no statistically significant difference between injured practitioners with more or less physical conditioning. As well as stretching, which is not performed by most of the practitioners (53.7%), before and after the games also did not present relevance as a risk factor. In a literature review available on these relations,^5^ we also did not find statistical relevance regarding the practice of muscle stretching before and after exercise and the decrease in injury risk, although they state that better physical conditioning, which is defined in the article^7^ as higher neuromuscular fiber recruitment, higher capacity of fibers to absorb energy and transfer it to the bone system, and higher amount of energy substrate is related to a lower incidence of injuries.

When the anthropometric factors such as weight, BMI, age, and sex were evaluated, we found significant relevance only for age, and after 50 years 61.8% of the patients had injuries; the other data showed no statistical relevance as risk factors or prevention of possible injuries. We also did not find in other studies with throwing sports, such as handball,^6^ significant relevance for such relations.

Most (74.5%) of the BT practitioners also participate in other sports, but this data did not show statistical relevance as a risk or protection factor. The practice of weight-training, which in our study was not presented as a prevention factor for the appearance of injuries, is advocated for the prevention of injuries and for the improvement of physical conditioning in studies aimed at the correct practice of weight-training,^7^ where the authors argue that when well performed and supervised, strength training acts as a protection factor against musculoskeletal injuries due to the improvement of body perception, muscle and joint strengthening, and physical conditioning.

We tried to find out if BT practitioners with previous injuries would have greater chances of suffering a new injury, finding a positive relation for this. However the study was limited for not being able to relate the place of the previous injury with the new injury, not being possible to define if it occurred in an already fragile place or if it affected a different area.

## CONCLUSION

Among the practitioners of BT, those individuals older than 50 years, who practice the sport for a longer time, with a greater weekly frequency, for more hours in each game and who already presented a previous injury at the beginning of the practice are more prone to injuries, being able to infer that the greater exposure increases the risk of injury.

Actions that promote the adequate preparation and physical conditioning to the players of BT showed no difference in this study for the prevention of sports-related injuries and also in the time away from the game.

The prevalence of injuries showed no difference in relation to gender, nor to BMI.

The most common injuries were those of the elbow and shoulder, both due to inflammatory diseases of the tendons, followed by traumatic injuries (sprain) in the knee and ankle.
